# Case Report: Deficiency of Adenosine Deaminase 2 Presenting With Overlapping Features of Autoimmune Lymphoproliferative Syndrome and Bone Marrow Failure

**DOI:** 10.3389/fimmu.2021.754029

**Published:** 2021-10-14

**Authors:** Gianluca Dell’Orso, Alice Grossi, Federica Penco, Roberta Caorsi, Elena Palmisani, Paola Terranova, Francesca Schena, Michela Lupia, Erica Ricci, Shana Montalto, Filomena Pierri, Isabella Ceccherini, Francesca Fioredda, Carlo Dufour, Marco Gattorno, Maurizio Miano

**Affiliations:** ^1^ Hematology Unit, Istituto di Ricerca e Cura a Carattere Scintifico (IRCCS) Istituto Giannina Gaslini, Genoa, Italy; ^2^ Unitá Operativa Semplice Dipartimentale (UOSD) Genetics and Genomics of Rare Diseases, Istituto di Ricerca e Cura a Carattere Scintifico (IRCCS) Istituto Giannina Gaslini, Genoa, Italy; ^3^ Clinica Pediatrica e Reumatologia e Centro Malattie Autoinfiammatorie e Immunodeficienze, Istituto di Ricerca e Cura a Carattere Scintifico (IRCCS) Istituto Giannina Gaslini, Genoa, Italy; ^4^ Covid Hospital, Unità Operativa di Malattie Infettive, Dipartimento di Scienze Pediatriche, Istituto di Ricerca e Cura a Carattere Scintifico (IRCCS) Istituto Giannina Gaslini, Genoa, Italy; ^5^ Hematopoietic Stem Cell Transplantation Unit, Istituto di Ricerca e Cura a Carattere Scintifico (IRCCS) Istituto Giannina Gaslini, Genoa, Italy

**Keywords:** bone marrow failure (BMF), primary immune regulatory disorders (PIRDS), autoimmune lymphoproliferative syndrome (ALPS), next-generation sequencing (NGS), DADA2, inborn errors of immunity (IEI)

## Abstract

Deficiency of adenosine deaminase 2 (DADA2) is an autosomal recessive disease associated with a highly variable clinical presentation, such as vasculitis, inflammation, and hematologic manifestations. Some associations of clinical features can mimic autoimmune lymphoproliferative syndrome (ALPS). We report a case of a female patient who fulfilled the 2009 National Institute of Health revised criteria for ALPS and received a delayed diagnosis of DADA2. During her childhood, she suffered from autoimmune hemolytic anemia, immune thrombocytopenia, and chronic lymphoproliferation, which partially responded to multiple lines of treatments and were followed, at 25 years of age, by pulmonary embolism, septic shock, and bone marrow failure with myelodysplastic evolution. The patient died from the progression of pulmonary disease and multiorgan failure. Two previously unreported variants of gene ADA2/CECR1 were found through next-generation sequencing analysis, and a pathogenic role was demonstrated through a functional study. A single somatic STAT3 mutation was also found. Clinical phenotypes encompassing immune dysregulation and marrow failure should be evaluated at the early stage of diagnostic work-up with an extended molecular evaluation. A correct genetic diagnosis may lead to a precision medicine approach consisting of the use of targeted treatments or early hematopoietic stem cell transplantation.

## Introduction

Deficiency of adenosine deaminase type 2 (DADA2) is an autosomal recessive disease caused by loss-of-function mutations of the ADA2/CECR1 gene, which encodes adenosine deaminase type 2 (ADA2) (1). ADA2 is partially homologous to adenosine deaminase type 1 (ADA1) (1), which is involved in a key step of purine metabolism by breaking down adenosine (Ado) and 2′-deoxyadenosine (dAdo) to deoxyinosine (2, 3). However, ADA2 has a distinct 59-kDa structure and a lower affinity to Ado and dAdo, accounting for a limited role in purine metabolism and additional non-redundant functions. In fact, one type of adenosine deaminase cannot compensate for the absence of the other enzyme, as ADA1 deficiency results in severe combined immunodeficiency (1). Unlike ADA1, ADA2 forms homodimers with a molecular weight of ~110 kDa (3), and it is produced by activated monocytes, macrophages, and dendritic cells during inflammatory response, as in patients with an autoimmune disease or infections (1, 4–7). For proper translocation to extracellular space, ADA2 needs to be N-glycosylated (8). Upon release, ADA2 binds to the surface of various immune cells, possibly through the PRB domain (9), to induce the T-cell-dependent differentiation of monocytes into macrophages and a growth factor activity, which is partially unknown. ADA2 deficiency is associated with monocyte polarization to M1 macrophages, which are known to induce inflammation and tissue damage and increase the release of proinflammatory cytokines (1, 9, 10).

The clinical onset of DADA2 was reported before 1 and 10 years of age in 24 and 77% of patients, respectively, with a mortality rate of 8% before the age of 30 years. The clinical features of 161 patients have been retrospectively reported by Meyts et al. in 2018 (1), showing a highly variable and misleading clinical presentation due to vasculitis/vasculopathy of small- and medium-sized arteries. Skin manifestations were reported in >75% of patients, while neurological involvement with ischemic or hemorrhagic stroke was present in 50%, with potential underestimation when presenting as transient ischemic attacks (1). Consistent with a systemic inflammatory process, most patients experience recurrent fever, myalgia, arthralgia, serositis, and elevated inflammatory markers such as erythrocyte sedimentation rate and C-reactive protein (3). Less commonly, gastrointestinal and renal involvement, arthritis, and myositis were reported (11, 12). In addition to the mentioned inflammatory features, significant hematologic and immunologic involvement has been described recently. Hypogammaglobulinemia and a common variable immune deficiency (CVID) phenotype have been described in 25% of patients, with or without concurrent findings of vasculopathy (1, 3). Clonal lymphoproliferation (13), generalized lymphoadenopathy (>10%), and splenomegaly (up to 30%) were also reported. Other later reports described further hematological manifestations, including pure red cell aplasia (PRCA), and cytopenia affecting one or more cell lineages (12, 14). The specific association of symptoms might resemble autoimmune lymphoproliferative syndrome (ALPS), as described in a report by Alsultan (15). The severity of the marrow failure of the patient may lead to the indication of hemopoietic stem cell transplantation (HSCT), which represents the only curative option for congenital diseases (16). HSCT has been used in patients with a severe phenotype (10, 17–19) that did not respond to medical treatments such as tumor necrosis factor (TNF) inhibitors, which represent the best option in controlling fever episodes and vasculopathy and in preventing stroke (1, 20, 21).

We describe a case of a young woman with a long history of ALPS during childhood followed by rapid evolution to bone marrow failure, which resulted from carrying a novel pathogenic genotype of the ADA2/CECR1 gene.

## Case Presentation

**Figure 1:**
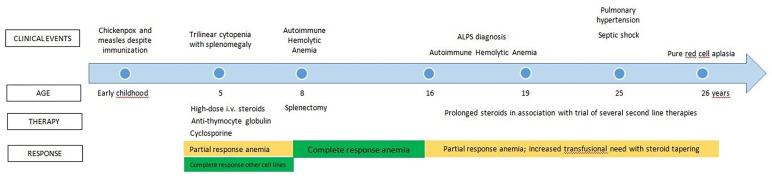
Clinical history previous to referral at our Center.

The clinical history of the patient is summarized in **Figure 1**. Apart from chickenpox and measles that occurred despite specific vaccination, no significant clinical issues were reported during early childhood. Another center followed up with her since the age of 5 years after an episode of trilinear cytopenia associated with splenomegaly. The marrow examination demonstrated good cellularity. No detailed information on therapeutic approaches was available at that time. Her family reported that she was treated with high-dose steroid therapy, transfusions, anti-thymocyte globulin, and cyclosporine A, with a complete recovery on platelet count and a partial response on other cell lines.

Three years later, an episode of acute autoimmune hemolytic anemia (AIHA) was successfully treated with splenectomy. At the age of 16, she developed several new episodes of AIHA associated with chronic lymphoproliferation and high values of T cell receptor αβ^+^ CD4^-^ CD8^-^ double-negative T cells (DNT). Defective Fas-mediated T-cell apoptosis was demonstrated in two different laboratories in order to obtain diagnostic confirmation. She received a diagnosis of ALPS, according to the 2009 National Institute of Health (NIH) revised criteria (22). Along with steroid treatment, she received other lines of therapy, such as cyclophosphamide, rituximab, micophenolate mofetil, azathioprine, vincristine, and, lastly, tacrolimus. All these therapeutic options, performed over about 10 years, only resulted in a partial response of steroid-dependent AIHA. In fact, attempts to withdraw steroids were followed by an increased transfusion need. At that stage, the bone marrow examination was still normal.

At the age of 25, during follow-up at the other center, she received a chest X-ray, which revealed a potential lung nodule that required further evaluations. A computed tomography scan and lung scintigraphy showed features of pulmonary embolism, leading to a diagnosis of pulmonary hypertension without any previous symptom or thrombotic event. Thrombophilia screening demonstrated protein S deficiency; therefore, apixaban prophylaxis was started. Meanwhile, she developed septic shock from *Streptococcus gallolyticus*, requiring intensive care. One year later, she was referred to our center for a second opinion because of worsening anemia despite the steroid and tacrolimus treatment.

**Table 1** shows the significant results of a blood examination performed on admission to our center. Hyporegenerative anemia and mild neutropenia were found. An immunological re-evaluation confirmed that her case fulfilled the 2009 NIH ALPS criteria, but with a significant reduction in immunoglobulin levels, and her plasma-soluble FAS ligand levels were normal. The trephyne biopsy showed severe erythroid hypoplasia, associated with normal myeloid/lymphoid cellularity and megakaryocytes. The marrow progenitor assay demonstrated reduced numbers of burst forming unit-erythroid and colony-forming unit for granulocytes and macrophages. The addition of the plasma of the patient to heterologous marrow cell precursors inhibited cellular growth and differentiation, possibly suggesting a humoral inhibitory effect on the marrow progenitor cells. Based on the clinical and laboratory findings and on the unsatisfactory control of the clinical symptoms, tacrolimus was substituted by sirolimus, while the steroids were slowly tapered off. Due to the absence of data on immunoglobulin levels before rituximab administration, it was not possible to determine whether hypogammaglobulinemia was either treatment- or disease-related, although the previously failed attempt to immunize against measles and chickenpox raised the suspicion of a previous CVID phenotype. Therefore, a program of regular subcutaneous immunoglobulin administration was started in order to reduce any risk of secondary infections related to the immunosuppressive treatment. Iron chelation treatment was also started due to elevated ferritin levels secondary to previous intensive transfusion support. Since sirolimus did not produce any response, erythropoietin was additionally administered weekly. At that stage, the patient was continuously offered HSCT, but it was strongly refused.

**Table 1:** Significant laboratory tests at admission in our center.

	Results	Reference range
Hemoglobin	9.2 g/dl	11.5–16.5
Lactic dehydrogenase	983 U/L	84–480
Haptoglobin	<2 mg/dl	15–160
Total lymphocyte count	7,150/mmc	3,600–9,800
Lymphocyte subsets		
CD3^+^TCRαβ^+^CD4^−^CD8^−^ DNT cells	5.1% (365/mmc)	<1.5% of total lymphocytes
B cells CD 19^+^	2.6% (186/mmc)	6–19%
B cells CD27^+^ (CD19^+^ tot)	15.9% (1,137/mmc)	>15%
CD3CD25^+^/CD3HLADR^+^ ratio	0%	>1
CD3^+^TCR αβ^+^ CD4^−^CD8^−^ B220^+^ cells^a^	82.7% (5,913/mmc)	<60%
Autoimmune lymphoproliferative syndrome (ALPS) biomarkers		
Plasma sFASL levels	0.5 pg/ml	0 >200 in ALPS
Elevated plasma interleukin-10 levels	<1 pg/ml	<1 pg/ml >20 in ALPS
Elevated serum or plasma vitamin B12 levels	374 ng/L	191–663 >1,500 in ALPS
Elevated plasma interleukin-18 levels	5,750 pg/ml	36–258 >500 in ALPS
Immunoglobulin G	254 mg/dl	700–1600
Immunoglobulin A	13 mg/dl	70–400
Immunoglobulin M	299 mg/dl	40–230

a Increased B220+ T lymphocytes are significantly associated with ALPS with good specificity (23–25).

At 5 months after being referred to our center, the patient developed severe neutropenia and fever, requiring hospitalization. The trephine biopsy demonstrated severely reduced granulocytopoiesis and erythropoiesis and dysmegakaryocytopoiesis. The patient quickly developed an overwhelming hyperinflammatory syndrome and, due to the progressive worsening of her respiratory function, she was admitted to the intensive care unit. Unfortunately, despite extracorporeal membrane oxygenation, the patient died from progressive multiorgan failure and right ventricular cardiac thrombosis.

## Diagnostic Assessment

**Figure 2:**
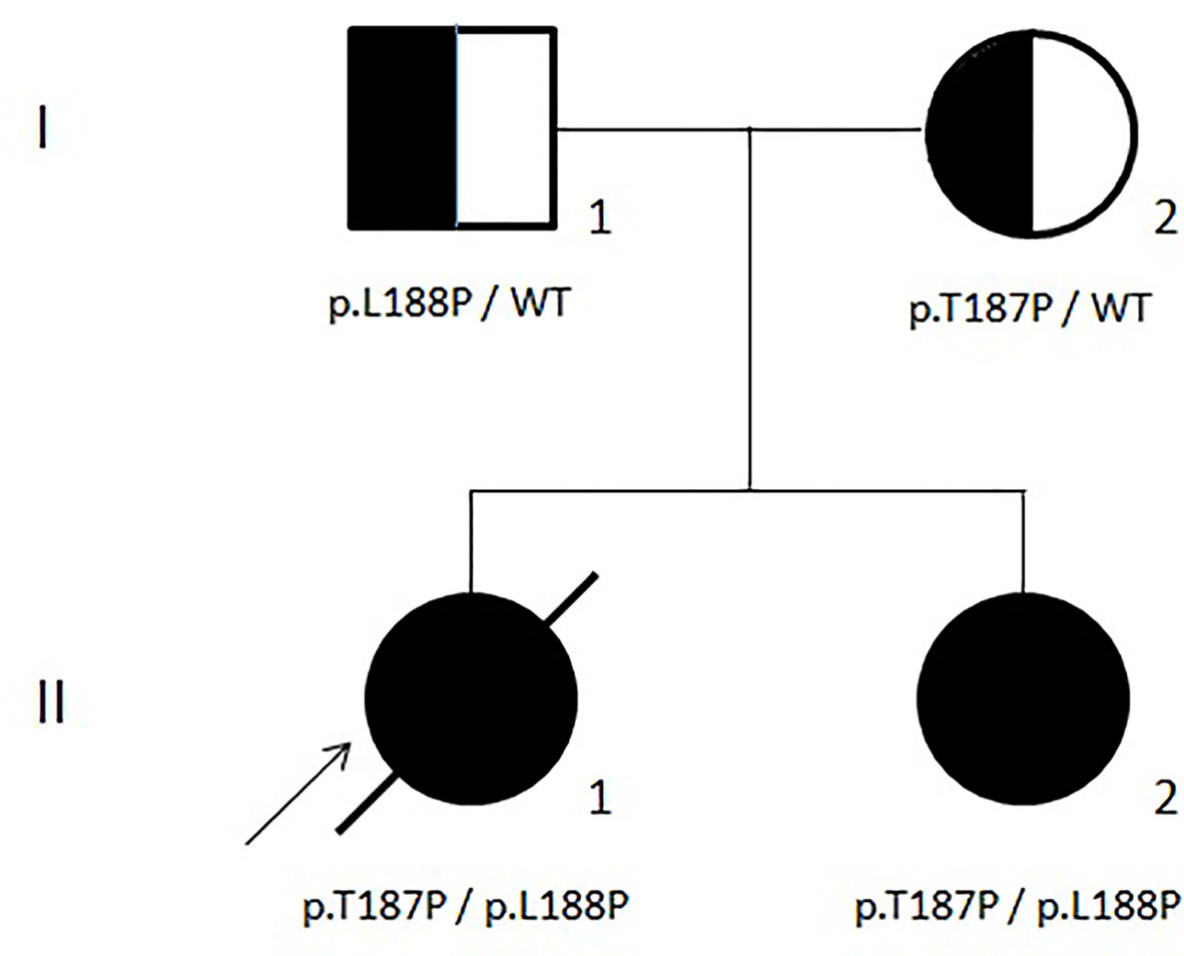
Family tree.

During the follow-up at our center, a next-generation sequencing (NGS) panel that included genes related to both congenital marrow failure and immune dysregulation syndromes (26, 27) was applied to our proband (II-1 in **Figure 2**). Unfortunately, the results were released only a few days before the death of the patient and showed two germline mutations of the ADA2/CECR1 gene (OMIM#607575; transcript NM_001282225.2): (i) c.563T>C, leading to p.Leu188Pro, reported also by Michniacki in 2018 in association with DADA2 (28), and (ii) c.559A>C, leading to p.Thr187Pro, previously unreported. Based on the American College of Medical Genetics and Genomics criteria (29), both variants are classified as having a “likely pathogenic” effect and, consistent with the autosomal recessive inheritance of DADA2 (OMIM#615688), they turned out to be inherited by her father and mother, respectively (**Figure 2**). These observations confirmed the causal role of the ADA2/CECR1 genotype of our patient on her condition. Both mutations were also found in the sister of the patient (II-2 in **Figure 2**), who displayed a clinical history of polyarticular arthritis of the small joints of the hands, along with Raynaud’s phenomenon, hip and knee arthralgia, mild leukopenia, and mild thrombocytopenia. After the result of the genetic test, a targeted immunological screening revealed hypogammaglobulinemia and increased values of DNT cells (2.5%) in the mother of the patient (I-2 in **Figure 2**).

**Figure 3:**
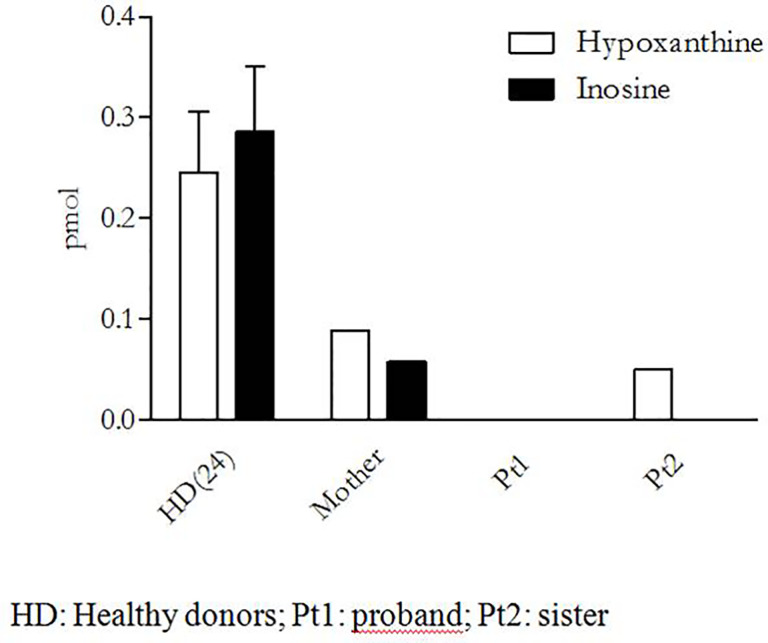
Functional assay of ADA2 activity.

Since none of the ADA2/CECR1 variants found in our patient were reported in any database of pathogenic mutations at the moment of her genetic diagnosis, a functional analysis on peripheral monocytes was performed to test their effect on ADA2 activity. These cells were isolated by adherence, after peripheral blood mononuclear cell Ficoll–Paque separation, and were then cultured in phosphate-buffered saline with exogenous adenosine (Sigma Aldrich) with or without ADA1 inhibitor erythro-9-(2-hydroxy-3-nonyl) adenine (Sigma Aldrich) for 4 h at 37°C with 5% of CO_2_. The supernatants were collected, and the activity enzyme was indirectly evaluated in high-performance liquid chromatography through the measurement of the adenosine-derived products (inosine and hypoxanthine) as a surrogate marker of enzyme activity (20). As shown in **Figure 3**, no adenosine metabolites were detectable in our patient (Pt 1), thus suggesting a complete loss of enzymatic activity. Consistently, both the patient and her sister presented compound heterozygosity for the same variants, and we could demonstrate a complete absence of inosine, the most important adenosine-derived product.

Finally, a heterozygous pathogenic somatic mutation on STAT3 (p.Lys658Arg) was also identified in our patient. The same mutation was found neither in her parents nor in her sister, and its somatic origin was confirmed by its absence in the skin fibroblasts of the patient.

## Discussion

The clinical history of the patient was characterized by symptoms and laboratory findings fulfilling the 2009 NIH ALPS diagnostic criteria (22), followed, in early adulthood, by the onset of more specific features of DADA2, such as vasculopathy, marrow failure, and hyperinflammatory symptoms (1).

ADA2/CECR1 missense, frameshift mutations, splicing defects, or deletions have been described as pathogenic and were distributed in all different structural domains (3, 11, 30, 31). In a recent work, Lee et al. performed a literature review and a genotype comparison of vasculitis and hematologic phenotypes in DADA2. In the manuscript, the ADA2/CECR1 mutations were clustered in groups according to their predicted residual enzymatic activity. The prevalence of PRCA or marrow failure features was greater in groups according to their lower predicted enzymatic activity (<3% residual enzymatic activity), in particular, with insertion–deletion mutations (indels), early-termination mutations, and missense mutations, including Leu188Pro, which we found in our patient (32). However, the pathogenic mechanism of the residual enzymatic activity toward vasculitis or marrow failure remains to be determined.

The two novel ADA2/CECR1 mutations found in our case could explain both the ALPS and DADA2 phenotypes.

Unusual phenotypes with features overlapping both rheumatological and hematological disorders have been already reported not only in DADA2 patients but also in other autoinflammatory/autoimmune disorders (23), which can show the expansion of DNT cells and other ALPS markers, making the diagnosis particularly challenging. Similarly, a significant proportion of ALPS patients may also present with a consistent inflammatory phenotype (23).

In the first phases of the disease, the patient fulfilled the NIH 2009 ALPS criteria (22). However, some typical ALPS biomarkers, such as sFAS, IL-10, and vitamin B12, resulted to be normal. The immunoglobulin levels in this patient were not a reliable diagnostic criterion due to a previous rituximab treatment (33). In addition, although anemia was initially secondary to peripheral autoimmune hemolysis with normal marrow cellularity, in the following years, it became hyporegenerative with erythroid hypoplasia and tested negative in both direct and indirect antiglobulin tests, a feature atypical of ALPS. Over the past 10–15 years, improvements in genomic technologies have led to the description of a number of monogenic disorders mimicking ALPS. These rare conditions, defined as CVID or ALPS-like phenotypes, clinically resemble ALPS and, therefore, are often misdiagnosed, highlighting the urgent need to revise the NIH ALPS diagnostic criteria based on increased knowledge of the pathogenic mechanisms and biomarkers of such disorders (23–25, 34, 35). Therefore, an earlier genetic diagnosis should be performed in all patients with immune dysregulation to define a more precise therapeutic strategy and to make a proper assessment in case of stem cell transplantation. The most important signal for correctly diagnosing and treating this patient was the progressive evolution of the clinical phenotype over time, with prevalent inflammatory features, vasculitis, and bone marrow failure with PRCA, although such signs and symptoms of DADA2 and the disease itself were still mostly unknown at that time.

In our patient, marrow involvement, initially characterized by PRCA, evolved into severe trilinear marrow failure, in keeping with the concept that DADA2 phenotypes likely represent a continuum rather than different categories (32).

A colony-forming unit assay clearly showed not only the reduced growth of marrow progenitor cells but also an inhibitory effect on the plasma of the patient with heterologous marrow progenitors, suggesting a potential contribution of humoral immunity possibly related to immune dysregulation. Indeed the pathogenesis of marrow failure in ADA2 deficiency remains largely not understood. An ADA2 knocked-down zebrafish model displays neutropenia, thus supporting an intrinsic role of ADA2 in normal hematopoiesis (12). On the other hand, human ADA2 was shown to have an *in vitro* growth factor activity (7) whose absence may have contributed to the development of marrow failure.

In addition, the coexistence of strongly diminished ADA2 activity with an oligosymptomatic phenotype in the sister can be explained by well-known intrafamilial phenotypic variability despite the same underlying homozygous mutations (11,19,30,36–40). However, even if individuals with biallelic ADA2/CECR1 pathogenic variants were reported to have remained asymptomatic until adulthood or to have never developed clinical manifestations of DADA2 (41), the sister of our patient is currently following up with another adult rheumatology center.

A gain-of-function, likely pathogenic somatic heterozygous STAT3 somatic mutation, was also shown by the NGS panel in the marrow cell of our patient. This variant had not been previously reported. The STAT3 gene (42) encodes a transcription factor activated in response to cytokine signaling, and germline gain-of-function STAT3 mutations were reported after whole-exome sequencing and whole-genome sequencing studies as new potential genetic drivers of ALPS-like phenotypes (43, 44). On the other hand, somatic heterozygous STAT3 gain-of-function mutations are also reported in literature in association with myelodysplastic syndrome (45–47). We found this mutation only in cells derived from the hematopoietic lineage, while skin fibroblasts resulted as wild type for STAT3. Unfortunately, it is not possible to define the contribution of the STAT3 mutation in our patient due to the unavailability of marrow samples and genetic tests at the onset of her symptoms. We can only speculate that such mosaicism might have been either a sign of a myelodysplastic evolution or present since diagnosis, contributing to the onset of the ALPS phenotype, similar to somatic mutations in the FAS gene (48, 49).

The overlap between marrow failure and immune dysregulation has recently been documented by our group in a large study cohort of patients (27). This reinforces the idea that young patients with marrow failure should undergo early immunological screening and be offered genetic tests by either extended next-generation sequencing panels (50), which include genes leading to primary immune deficiencies, or unbiased whole-exome sequencing, when available. In fact, improvements in diagnostic accuracy may lead to an early targeted therapy. In our patient, an earlier diagnosis of DADA2 could have led to a more prompt and tailored treatment with anti-TNF alpha, potentially improving the inflammatory phenotype and controlling the progression of the disease (1, 21, 32). The previous indication to splenectomy could have been further evaluated, balancing rewards and risks as infectious risk, if a genetic diagnosis was available at that moment. She experienced an episode of sepsis and a hyperinflammation evolving in fatal multiorgan failure with cardiac thrombosis: the association of splenectomy and several immunosuppressive treatments could have represented the risk factors for such complications. HSCT, even in the absence of a genetic diagnosis, could have prevented the fatal progression of other co-morbidities, but the patient strongly refused it. This procedure may be considered earlier for patients with severe hematologic presentation (10, 17–19, 32).

In conclusion, this case report suggests that clinical phenotypes encompassing immune dysregulation and marrow failure should be evaluated at the early stage of diagnostic work-up with an extended molecular evaluation that includes genes that cause both groups of disorders. Proper genetic diagnosis may lead to precision medicine approach and targeted treatments.

## Data Availability Statement

The datasets presented in this study can be found in online repositories. The names of the repository/repositories and accession number(s) can be found below: (https://www.ncbi.nlm.nih.gov/clinvar/) VCV000973671.1, VCV000973614.1, and VCV000421491.2.

## Author Contributions

GD and MM conceived the presented idea. GD, RC, EP, ER, SM, FiP, MG, CD, and MM reviewed the clinical information presented. MG, CD, and MM oversaw the writing, data collection, and editing process. IC, MG, FF, CD, and MM provided critical review of the manuscript. PT and ML performed immunological assays. AG and IC performed genetic diagnosis. FeP, RC, and FS performed functional assay on a research basis. All authors contributed to the article and approved the submitted version.

## Conflict of Interest

The authors declare that the research was conducted in the absence of any commercial or financial relationships that could be construed as a potential conflict of interest

## Publisher’s Note

All claims expressed in this article are solely those of the authors and do not necessarily represent those of their affiliated organizations, or those of the publisher, the editors and the reviewers. Any product that may be evaluated in this article, or claim that may be made by its manufacturer, is not guaranteed or endorsed by the publisher.
